# MIxS-HCR: a MIxS extension defining a minimal information standard for sequence data from environments pertaining to hydrocarbon resources

**DOI:** 10.1186/s40793-016-0203-5

**Published:** 2016-10-12

**Authors:** Nicolas Tsesmetzis, Pelin Yilmaz, Peter C. Marks, Nikos C. Kyrpides, Ian M. Head, Bart P. Lomans

**Affiliations:** 1 Shell International Exploration and Production Inc., 3333 HW6S, Houston, 77082 TX USA; 2 Microbial Genomics and Bioinformatics Research Group, Max Planck Institute for Marine Microbiology, Bremen, Germany; 3 DOE Joint Genome Institute, Walnut Creek, CA 94598 USA; 4 School of Civil Engineering and Geosciences, Newcastle University, Newcastle upon Tyne, NE1 7RU UK; 5 Shell Global Solutions International B.V., Rijswijk, Netherlands

**Keywords:** Hydrocarbon resources, Sequence data, MIxS standards, Environmental package

## Abstract

Here we introduce a MIxS extension to facilitate the recording and cataloguing of metadata from samples related to hydrocarbon resources. The proposed MIxS-HCR package incorporates the core features of the MIxS standard for marker gene (MIMARKS) and metagenomic (MIMS) sequences along with a hydrocarbon resources customized environmental package. Adoption of the MIxS-HCR standard will enable the comparison and better contextualization of investigations related to hydrocarbon rich environments. The insights from such standardized way of reporting could be highly beneficial for the successful development and optimization of hydrocarbon recovery processes and management of microbiological issues in petroleum production systems.

**Electronic supplementary material:**

The online version of this article (doi:10.1186/s40793-016-0203-5) contains supplementary material, which is available to authorized users.

## Background

**Fig. 1:**
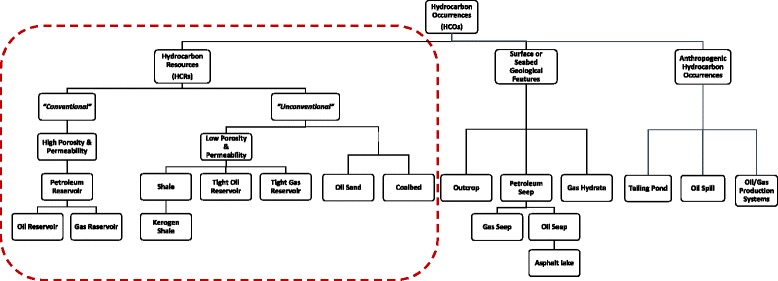
Hydrocarbon Resources as part of the Hydrocarbon Occurrences environmental features. Schematic of the break-down of Hydrocarbon Occurrences (HCOs) based on location and commercial exploitation potential. The hydrocarbon resources (HCRs), shown in the dotted frame, are further subdivided into different groups based on their associated reservoir rock properties and hydrocarbon type

Hydrocarbon Occurrences are defined as the natural and artificial environmental features that are rich in hydrocarbons (Fig. 1). Hydrocarbon Occurrences that can be exploited in a commercially viable manner are designated as Hydrocarbon Resources (dotted frame in Fig. 1). HCRs currently cover over 80 % of our global energy needs and they will continue to do so through 2040 [1]. Contrary to the public perception, these hydrocarbon-rich environments are often inhabited by microorganisms. In situ processes including oil degradation, methane generation, and hydrogen sulfide production are to a great extent driven or accelerated by the activity of microorganisms in these systems [2]. Moreover, microorganisms are also implicated in metal corrosion and fouling of the hydrocarbon production and transport infrastructure [2]. If left uncontrolled, such microbial (or microbially influenced) processes can lead to adverse environmental and operational consequences. On the other hand, novel applications where microbes can play a more positive role in petroleum systems are becoming increasingly evident. These include hydrocarbon exploration, reservoir souring prevention, hydrocarbon upgrading and enhanced hydrocarbon and energy recovery.

Restraining or harnessing the potential of these microbes requires a considerable understanding of their metabolisms and role in these environments. Tools and methodologies that allow the enumeration, taxonomic identification, and metabolic prediction of these microbes are therefore essential towards this goal. Such methodologies will enable us to address questions relating to microbial spatiotemporal variability and what drives them within an HCR. This will also help us determine whether particular microorganisms were present in the HCR at the time of deposition or whether they were introduced at a later stage through fluid migration (e.g. during the formation or recovery of the hydrocarbon resource). Other interesting questions pertain to hydrocarbon recovery processes, and especially the effect of water injection (aka “water flooding”) on the indigenous and introduced microbial communities associated with HCRs. One particular operational issue related to this is stimulation of biogenic H_2_S production, an undesirable process better known as reservoir “souring”. Moreover, in terms of microbial monitoring, such methodologies will help us determine to what extent the indigenous microbial community of an HCR is represented by the analysis of readily recoverable produced fluids, and if analysis of produced fluids can reduce the need to analyze material directly recovered from the hydrocarbon rich formation, such as reservoir cores which are much more difficult to obtain. It will also be practically important if the analysis of planktonic populations from produced fluids can provide an alert to potential MIC phenomena caused by sessile populations. A growing inventory of HCR systems will also allow us to determine if specific beneficial or detrimental microbially catalyzed processes in HCRs are the result of the same microbial populations in most instances, or whether a particular process is driven by different organisms in different HCR environments.

In addition to environmental parameters, the outcome from microbiological surveys of HCRs can be strongly impacted by logistical and technical constraints. These include sample acquisition, transport and preservation; the choice of DNA amplification primers (i.e. coverage, specificity, target gene); enumeration methods (i.e. MPN vs. ATP vs. qPCR vs. Phylochip vs. Next-Gen sequencing, etc.); sequencing platform (Sanger, 454, IonTorrent, Illumina, etc.) and related downstream bioinformatics pipelines.

### Need for standards

There is a growing list of studies on HCRs, but their collective retrieval (or their corresponding sequences from public repositories) is currently impossible even with complex keyword searches. Moreover, the majority of available datasets lack sufficient contextual data, which would facilitate more comprehensive comparative analysis [3–6].

In order to maximize the knowledge gained from these largely unexplored microbial ecosystems it is important to formalize and standardize environment descriptors for studies of these habitats. It is equally important to define a minimum set of contextual parameters, which should accompany the submission of sequence information from HCR studies to the International Nucleotide Sequence Database Collaboration [7]. The adoption of such standardization would drastically improve the quality, accessibility and value of the HCR-related information residing in INSDC.

The need for standardization is not novel or unique to HCR studies. Since 2005, the Genomic Standards Consortium [3, 4] has made remarkable efforts in proposing standards for genomic (MIGS) and metagenomic (MIMS) sequences [8, 9] as well as for marker genes (MIMARKS) [10] and biosynthetic gene clusters (MIBiG) [11]. Moreover, a single entry point to all the minimum information specifications was also proposed (Minimum Information about any Sequence; MIxS) [10]. Equally important, the GSC proposed a wide range of environmental packages, which cover a broad range of the commonly encountered environments in research studies (i.e. human- associated, soil, water, sediments, built environments, etc.) [10, 12].

Around the same time a separate effort aimed at the development of ENVO, a standardized and semantically controlled representation of environment descriptors was undertaken [13]. ENVO quickly became a core component of the MIxS specification. Despite being extensively developed for other environmental features and habitats, ENVO currently has very limited content related to HCR. For example, in the MIxS specification under the ENVO term *biome* only the subclasses *aquatic biome*, *polar biome*, and *terrestrial biome* are currently present. The term *subterranean biome* (or *subterrestrial biome*), which would include biomes related to certain HCR (e.g. hydrocarbon reservoir) as well as other subterranean biomes [14–16] is currently missing. Similarly, in the case of ENVO’s *environmental feature* branch, additional HCR terms such as *gas reservoir*, *oil sand*, and *coalbed* will need to be included in addition to the existing *oil reservoir* term. Finally, in the *environmental material* section of ENVO, HCR terms like *formation water*, *injection water*, *drilling fluid, tailing pond* and many more would supplement the existing *oil field production water* term. It is therefore apparent that expansion of ENVO to include HCR-related terms will greatly benefit the standardization of a growing number of studies on these environments. An initiative to introduce such HCR-related terms in ENVO is currently underway.

### Implementation of a Hydrocarbon Resource Environmental package

In an effort to assist with the standardization of data acquisition and observations derived from HCR-related environments we introduce the MIxS-HCR minimum information standard. This standard is tailored for HCR-related studies and aims at capturing key environmental parameters influencing microbial activity in these environments and standardizing their method of reporting. This is accomplished by the adoption of terms (such as temperature, pressure, porosity, etc.) from previously reported environmental packages (i.e. Water, Sediment, Wastewater/Sludge, etc.) as well as the introduction of new checklist items specific to these environments. A checklist consisting of 93 fields from several disciplines including geology, geochemistry, petrophysics, reservoir engineering, and production chemistry has been compiled (Table 1 and [MIxS HCR detailed table] in Additional file 1). Some of the included terms pertain to the HCR entity as a whole whereas others concern the sample(s) acquired from that entity. Moreover, the checklist is divided into 5 sections to facilitate the grouping of items derived from the same type of analysis or the same topic (Table 1). These sections include general information about the HCR, descriptors related to the HCR’s production history, the sample’s hydrocarbon and water chemistry, sampling procedures, sample transport and storage conditions.

**Table 1:** MIxS-HCR package items. More detailed description of the field names is provided in the Additional file 1 [see MIxS HCR detailed table]

MIxS-HCR package	
HCR general properties	
Hydrocarbon resource type	
Hydrocarbon type produced	
Basin name	
Field name	
Reservoir name	
Hydrocarbon resource original temperature	
Depth (TVDSS) of hydrocarbon resource temperature	
Hydrocarbon resource original pressure	
Depth (TVDSS) of hydrocarbon resource pressure	
Permeability	
Porosity	
Lithology	
Depositional environment	
Hydrocarbon resource geologic age	
Oil water contact depth	
Formation water salinity	
Sulfate in formation water	
Vfa in formation water	
Source rock kerogen type	
Source rock lithology	
Source rock depositional environment	
Source rock geologic age	
HCR production properties	
Production start date	
Production rate	
Water production rate	
Water cut	
Injection water fraction	
Secondary and tertiary recovery methods and start date	
Injection water breakthrough date of specific well	
Biocide administration	
Biocide administration method	
Chemical treatment	
Chemical treatment method	
Corrosion rate at sample location	
HCR sampling properties	
Sample well name	
Well identification number	
Sample type	
Sample subtype	
Sample collection point	
Temperature	
Pressure	
Sample true vertical depth subsea	
Sample measured depth	
Elevation	
Oxygenation status of sample	
Preservative added to sample	
Sample transport conditions	
Sample storage temperature	
Sample storage duration	
Sample storage location	
Sample vol., weight, or area swabbed for DNA extraction	
Organism count	
Organism count qPCR information	
HCR water chemistry	
pH	
Sample salinity	
Alkalinity	
Sulfate	
Sulfide	
Total sulfur	
Nitrate	
Nitrite	
Ammonium	
Total nitrogen	
Dissolved iron	
Sodium	
Chloride	
Potassium	
Magnesium	
Calcium	
Total iron	
Dissolved organic carbon	
Dissolved inorganic carbon	
Dissolved inorganic phosphorus	
Total phosphorus	
Suspended solids	
Density	
Dissolved carbon dioxide	
Dissolved oxygen	
Volatile fatty acids	
Benzene	
Toluene	
Ethylbenzene	
Xylene	
HCR hydrocarbon chemistry	
API gravity	
Total Acid Number	
Viscosity	
Pour point	
Saturates wt%	
Aromatics wt%	
Resins wt%	
Asphaltenes wt%	
Other properties	
Miscellaneous parameter	
Additional info	

Amongst the different minimal information standards mentioned above, the MIMS and MIMARKS survey sequence specifications are probably the most relevant ones for HCR-related studies as the majority of these studies involve single gene (i.e. 16S rRNA, dsr, nar, etc.) or whole metagenome surveys. As such, in addition to the HCR environmental package, the MIxS-HCR extension also includes a subset of the MIxS checklist containing MIMS and/or MIMARKS survey fields (depending on the study) (Additional file 1). This newly proposed MIxS-HCR minimum information standard provides the foundation for consistent capture and reporting of valuable contextual (i.e. environmental, biological and technical) information derived from HCR-related studies. An example of a MIxS-HCR- compliant report from a Brunei oil field is included in Additional file 1 [see MIxS HCR detailed table].

### Development process & research community

The need for standardization of HCR-related biological information has been the topic of several conference and workshops discussions where both academia and industry acknowledged the importance of adhering to standardized ways of sharing and reporting information. MIxS-HCR minimum information standard is the joint effort of a multidisciplinary community from academia and industry including the GSC MIxS developers, environmental microbiologists, bioinformaticians, geochemists, reservoir engineers, production chemists and computer scientists. During its development, the proposed HCR environmental package sought feedback and endorsement from researchers in academia and industry working in this scientific field. A web forum was set up to promote the development and refinement of the package as well as stimulate discussion around the topic and its content [17]. Changes to the package were subject to the consensus-based agreement amongst the researchers involved in this effort. The continuous contribution to the web forum and the adoption of this standard by the research community are key elements for the success of this initiative. Like its first release, which has already gained approval by the GSC board, yearly reviews of the MIxS-HCR standard performed by the MIxS-HCR web forum coordinators [18] will be incorporated in the next available MIxS public release following review and approval by the GSC board. As with all GSC projects, news and updates pertaining to this standard are managed via the corresponding project page on the GSC website [19] but also through the MIxS-HCR web forum [17]. The latest GSC-approved downloadable version of the MIxS-HCR extension is available under the GSC MIxS extensions webpage where additional information such as a list of terms, contact details and project information are also provided [20]. Each field in the supplied spreadsheet is accompanied by a definition, an expected value (including controlled vocabulary terms where applicable), the number of occurrences each field may be used, a value syntax, a preferred unit (if applicable) and other relevant recommendations (see also [MIxS HCR detailed table] in Additional file 1).

Endorsement of this MIxS-HCR minimum information standard by the GSC, strengthens the case for its incorporation by the INSDC in the list of prerequisites at the time of sequence submission. The MIxS-HCR minimum set will be complemented with other minimum sets (currently under development) describing hydrocarbon-rich environments which are not covered under the HCR term. These include anthropogenic hydrocarbon occurrences (e.g. oil and gas production systems) as well as surface and seabed hydrocarbon occurrences (e.g. cold seeps, outcrops, gas hydrates, etc.) (Fig. 1). Many of the MIxS-HCR fields are going to be shared across the different hydrocarbon occurrence types whereas new ones, specific for each of the other types, will also be proposed. Of particular importance will be the development of minimal information standards for sequence data from oil and gas production systems as these systems are allegedly subject to failures frequently attributed to MIC [21] raising environmental, safety and operational concerns.

## Conclusions

The newly proposed MIxS-HCR minimum information standard provides the foundation for consistent capture and reporting of valuable contextual information derived from studies pertained to hydrocarbon resources. Its first release has already gained approval by the GSC board and will be incorporated in INSDC’s sequence submission process. A web forum has also been set up to promote MIxS-HCR future improvements and extension to cover a wider range of hydrocarbon occurrences. Active involvement of the research community and adoption of the MIxS-HCR standard are key elements to the success of this initiative.

## Additional file

Additional file 1: MIxS HCR detailed table. Detailed description of the MIxS-HCR fields including terms, definitions, field requirements, syntax and examples. (XLSX 43 kb)

## Abbreviations

AHO: Anthropogenic hydrocarbon occurrences
ATP: Adenosine triphosphate
ENVO: Environmental ontology
GSC: Genomics standards consortium
HCOs: Hydrocarbon occurrences
HCRs: Hydrocarbon resources
INSDC: International nucleotide sequence database collaboration
MIBiG: Minimal information about a biosynthetic gene cluster
MIC: Microbially influenced corrosion
MIMARKS: Minimal information about a marker gene sequence
MIMS: Minimal information about a metagenomic sequence
MIxS: Minimal information about any sequence
MPN: Most probable number
qPCR: Quantitative polymerase chain reaction
SHO: Surface and seabed hydrocarbon occurrences
